# HMGCS2 enhances invasion and metastasis via direct interaction with PPARα to activate Src signaling in colorectal cancer and oral cancer

**DOI:** 10.18632/oncotarget.13006

**Published:** 2016-11-01

**Authors:** Shih-Wen Chen, Chiang-Ting Chou, Cheng-Chi Chang, Yue-Ju Li, Szu-Ta Chen, I-Ching Lin, Sang-Heng Kok, Shih-Jung Cheng, Jang-Jaer Lee, Tai-Sheng Wu, Min-Liang Kuo, Been-Ren Lin

**Affiliations:** ^1^ Graduate Institute of Oral Biology, School of Dentistry, National Taiwan University, Taipei, Taiwan; ^2^ Department of Nursing, Division of Basic Medical Sciences, Chang Gung University of Science and Technology, Chiayi Campus, Chiayi, Taiwan; ^3^ Chronic Diseases and Health Promotion Research Center, Chang Gung University of Science and Technology, Chiayi Campus, Chiayi, Taiwan; ^4^ Graduate Institute of Clinical Dentistry, School of Dentistry, National Taiwan University, Taipei, Taiwan; ^5^ Angiogenesis Research Center, National Taiwan University, Taipei, Taiwan; ^6^ Department of Dentistry, National Taiwan University Hospital, College of Medicine, National Taiwan University, Taipei, Taiwan; ^7^ Department of Medical Research, China Medical University Hospital, China Medical University, Taichung, Taiwan; ^8^ Department of Biotechnology, Asia University, Taichung, Taiwan; ^9^ Department of Pediatrics, National Taiwan University Hospital, Taipei, Taiwan; ^10^ Graduate Institute of Toxicology, College of Medicine, National Taiwan University, Taipei, Taiwan; ^11^ School of Medicine, Kaohsiung Medical University, Kaohsiung, Taiwan; ^12^ Department of Family Medicine, Changhua Christian Hospital, Changhua, Taiwan; ^13^ School of Medicine, Chung Shan Medical University, Taichung, Taiwan; ^14^ Division of Oral and Maxillofacial Surgery, Department of Dentistry, National Taiwan University Hospital, Taipei, Taiwan; ^15^ Department of Dentistry, National Taiwan University Hospital, College of Medicine, National Taiwan University, Taipei, Taiwan; ^16^ Department of Surgery, National Taiwan University Hospital, College of Medicine, National Taiwan University, Taipei, Taiwan; ^17^ Graduate Institute of Medicine, College of Medicine, Kaohsiung Medical University, Kaohsiung, Taiwan

**Keywords:** HMGCS2, PPARα, metastasis, CRC, OSCC

## Abstract

Mitochondrial 3-hydroxy-3-methylglutaryl-CoA synthase (HMGCS2) is the rate-limiting enzyme of ketogenesis. Growing evidence indicates that HMGCS2 may be involved in cancer progression, but its exact role is largely unknown. In this study, we demonstrate that *HMGCS2* mRNA expression is associated with poor clinical prognosis and outcomes in patients with colorectal cancer (CRC) and oral squamous cell carcinoma (OSCC). *In vitro*, ectopic expression of HMGCS2 enhanced cancer cell motility in a ketogenesis-independent manner. Moreover, HMGCS2 promoted Src activity by directly binding to peroxisome proliferator-activated receptor alpha (PPARα), a transcriptional activator of Src. Taken together, these results suggest that HMGCS2 may serve as a useful prognostic marker and vital target for future therapeutic strategies against advanced cancer.

## INTRODUCTION

Cancer is a major age-related disease worldwide. Each year, over 575,000 people die of cancer, and more than 1.5 million people are diagnosed [1–2]. Colorectal cancer (CRC) is one of the most common cancers and has an overall 5-year survival rate of only approximately 55% [3–4]. A contributing factor of this poor prognosis of CRC is its propensity to invade adjacent tissues and metastasize to distant organs [5–7]. Oral squamous cell carcinoma (OSCC) is the fourth most common cancer worldwide. In Taiwan, OSCC is the most common cancer in men, with nearly 5400 new cases and 2200 deaths per year. Despite advances in systemic therapies, the 5-year survival rate of OSCC has not improved over the past four decades [8–9]. For patients with recurrent disease or distant metastases, treatment options are more limited and overall survival is less than 1 year [10]. Therefore, elucidating novel mechanisms underlying CRC and OSCC progression and developing potential treatment strategies are both major priorities in cancer research.

Emerging evidence indicates that fundamental differences exist between the metabolic pathways of normal and malignant cells [11–13]. In contrast to normal cells, which derive most of their usable energy through oxidative phosphorylation, cancer cells depend heavily on substrate phosphorylation pathways to meet energy demands [14–16]. Ketogenesis is a crucial alternative metabolic pathway that provides lipid-derived energy for various organs during carbohydrate deprivation such as in fasting [17]. Ketone bodies may be vital fuel in ketogenesis for tumor initiation or metastasis. Therefore, ketone bodies are a potential high-energy resource that can enable a tumor to grow even when cut off from a blood supply [18, 19]. Mitochondrial 3-hydroxy-3-methylglutaryl-CoA synthase (HMGCS2), part of the HMG-CoA family of proteins, is the rate-limiting enzyme that catalyzes the first reaction in ketogenesis. Mutations in this gene are associated with HMG-CoA synthase deficiency [20]. Recent studies have provided evidence that several proteins in the ketogenesis pathway, including HMGCS2, acetyl-CoA acetyltransferase (ACAT1), D-hydroxybutyrate dehydrogenase (BDH1), 3-hydroxy-3-methylglutaryl-CoA lyase (HMGCL), and 3-ketoacid-coenzyme A transferase 1 (OXCT1), were upregulated in prostate cancer cells [21]. However, the roles of HMGCS2 in OSCC and CRC are not currently known.

In this study, we hypothesized that HMGCS2 may enhance cell invasion and metastasis during CRC and OSCC progression. We demonstrate that HMGCS2 functions as a transcriptional factor that binds to peroxisome proliferator-activated receptor alpha (PPARα), resulting in Src expression and activation in a metabolically independent manner.

## RESULTS

### *HMGCS2* mRNA expression is positively correlated with CRC and OSCC patients’ TNM stage, survival rate, and lymph node metastasis

**Figure 1:**
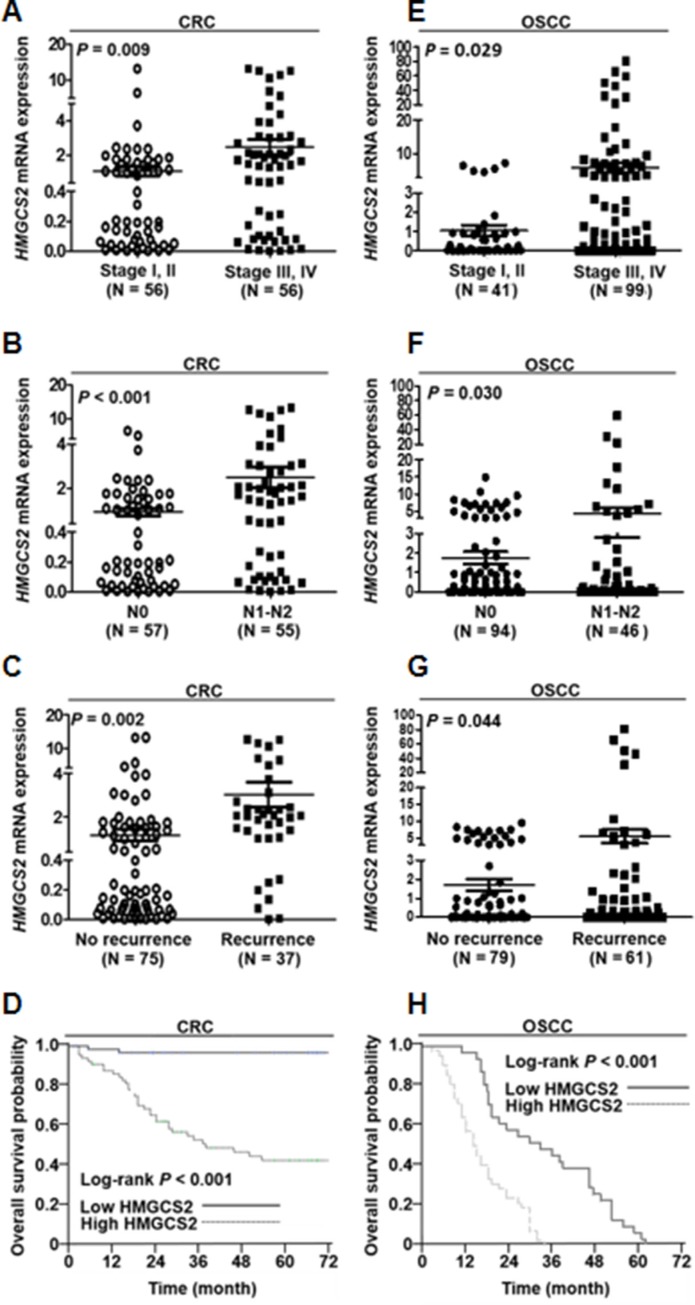
*HMGCS2* expression is positively correlated with TNM stage, survival rate, and lymph node metastasis in CRC and OSC patients (**A**–**C**) Real-time quantitative RT-PCR was performed on CRC patients’ tumors. Of the 112 CRC patients analyzed, the distributions of demographic, clinical, and pathological features are presented. (**D**) Patients were divided into high (fold change > cutoff values) or low (fold change ≤ cutoff values) HMGCS2 expression categories. Kaplan–Meier survival curves show that patients with low HMGCS2 expression (*n* = 55) survived significantly longer than those with high HMGCS2 expression did (*n* = 57; *P < 0.001). (**E**–**G**) Real-time quantitative RT-PCR was performed on OSCC patients’ tumors. Of the 140 OSCC patients analyzed, the distributions of demographic, clinical, and pathological features are presented. (**H)** Survival curves show that patients with low HMGCS2 expression survived significantly longer than those with high HMGCS2 expression did (*P* < 0.001).

To examine the clinical relevance of *HMGCS2* mRNA in patients with CRC, tissue was collected from both human CRC (*n* = 112) and OSCC (*n* = 140) tumors, and real-time quantitative RT-PCR analysis was performed. In CRC, higher *HMGCS2* mRNA expression levels were significantly associated with advanced TNM staging (*P* = 0.009, Figure 1A) and lymph node metastasis (*P* < 0.001, Figure 1B). Furthermore, *HMGCS2* mRNA expression was significantly higher in CRC patients with recurrence (*P* = 0.002, Figure 1C). To further clarify the correlation between postoperative patient survival and *HMGCS2* expression, we defined cutoff values as fold changes > 0.978 of baseline *HMGCS2* mRNA levels, as calculated through receiver-operating characteristic analyses, according to the most accurate predictive probability. On the basis of these criteria, the patients were divided into high (fold change > cutoff values) or low (fold change ≦ cutoff values) populations. Kaplan–Meier survival curves showed that CRC patients with low *HMGCS2* expression (*n* = 55) survived significantly longer than did those with high *HMGCS2* expression (*n* = 57; *P* < 0.001; Figure 1D). The cumulative 5-year survival rate for patients with low *HMGCS2* expression was 95.1%, whereas that for those with high *HMGCS2* expression was only 38.2%.

We also identified the clinical relevance of *HMGCS2* in OSCC. Higher *HMGCS2* mRNA expression levels were significantly associated with advanced TNM staging (*P* = 0.029, Figure 1E), lymph node metastasis (*P* = 0.030, Figure 1F), and recurrence (*P* = 0.0014, Figure 1G) in patients with OSCC. Survival curves showed that patients with low HMGCS2 expression survived significantly longer than did those with high HMGCS2 expression in OSCC (*P* < 0.001, Figure 1H).

To further examine the HMGCS2 mRNA expression in oral and colon normal tissue, Q-PCR was performed. The sample of adjacent normal tissue was collected, and the results demonstrated that *HMGCS2* mRNA expression was significantly lower in the portion of normal tissue compared to cancer part in OSCC and CRC (Supplementary Figure 1. CRC: *P* = 0.042, OSCC: *P* = 0.037). Taken together, our data suggest that elevated HMGCS2 mRNA expression is associated with advanced disease and poor outcomes in CRC and OSCC patients.

### HMGCS2 enhances cell migration and invasion abilities in CRC and OSCC cells

**Figure 2:**
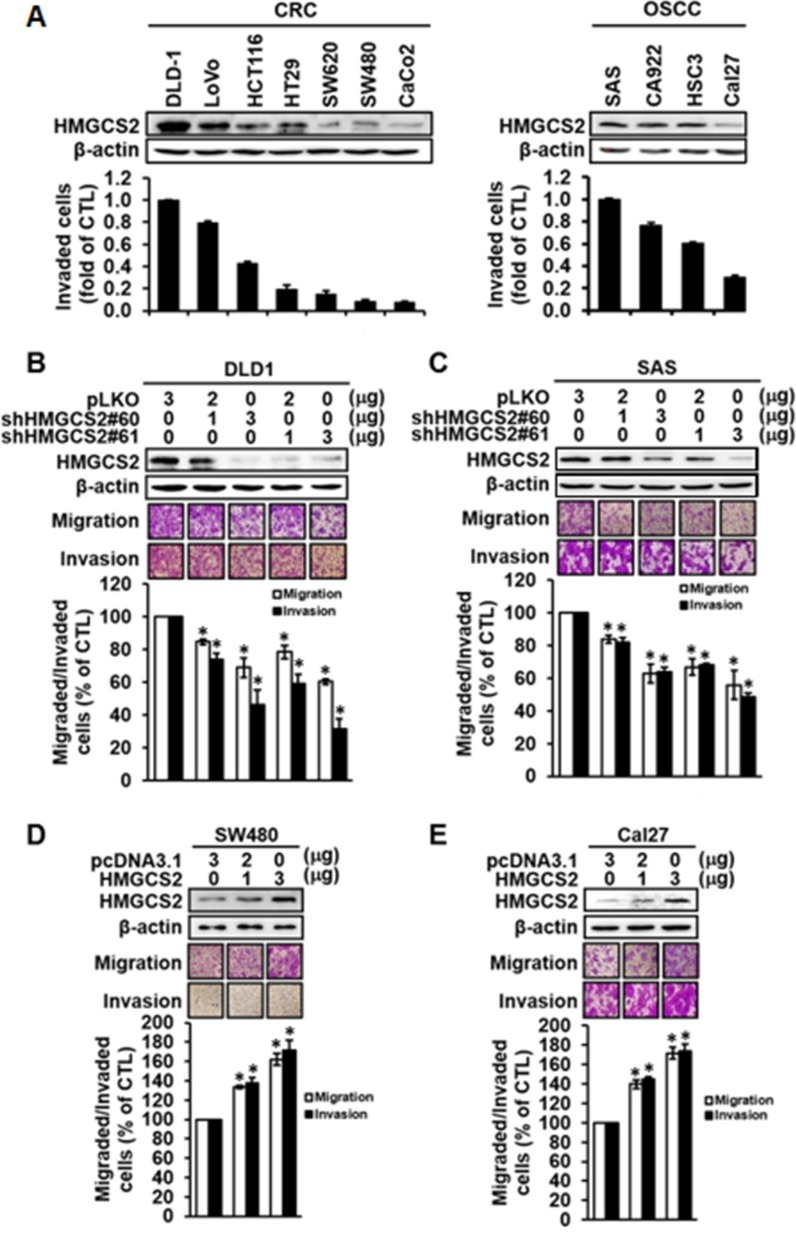
Overexpression and shRNA knockdown of HMGCS2 affect cell migration and invasion abilities in CRC and OSCC cells (**A**) Western blot analysis of endogenous HMGCS2 protein expression in CRC and OSCC cell lines. β-actin was used as an internal loading control (upper panel). The Boyden chamber assay was used to evaluate the invasion ability of CRC and OSCC cell lines (lower panel). (**B** and **C**) Cells were transiently transfected with control plasmids or various dosages of shHMGCS2 expression plasmids (upper panel). The Boyden chamber assay was used to evaluate the migration and invasion ability and number of migratory cells in DLD1 and SAS after transient knockdown of HMGCS2. Quantification of migratory cell number in DLD1 and SAS cells treated with shHMGCS2 expression plasmids (lower panel; *P < 0.05; **P < 0.001). (**D** and **E**) Cells were transiently transfected with control plasmids or various dosages of HMGCS2 expression plasmids (upper panel). Boyden chamber assay was used to evaluate the migration and invasion ability of migratory cells in SW480 and Cal27 after transient overexpression of HMGCS2. Quantification of the migratory cell number in SW480 and Cal27 cells treated with HMGCS2 expression plasmids (lower panel; *P < 0.05).

To study the roles of HMGCS2 in cancer progression, we first examined how its endogenous expression in wild-type CRC and OSCC cell lines correlates with cell motility. Figure 2A demonstrates that HMGCS2 protein expression was positively correlated with invasion ability in CRC and OSCC cell lines (left and right, respectively). HMGCS2 protein was highly expressed in advanced invasive cell lines, such as DLD-1, LoVo, SAS, and CA922, and expressed at lower levels in less invasive cell lines including SW480, Caco-2, and CAL 27. Transiently knocking down HMGCS2 with shHMGCS2 plasmids (#60 and #61) in DLD1 and SAS cells resulted in a dose-dependent decline in migrating and invading cells (Figure 2B, 2C). Ectopic expression of HMGCS2 in SW480 and Cal27 cells resulted in an enhancement of cell migration and invasion activities (Figure 2D, 2E). Notably, HMGCS2 did not increase proliferation in CRC and OSCC (Supplementary Figure 2). Taken together, these results indicate that HMGCS2 may increase cell motility in OSCC and CRC models.

### Stable knockdown of HMGCS2 reduces migration and invasion ability in CRC and OSCC cells

**Figure 3:**
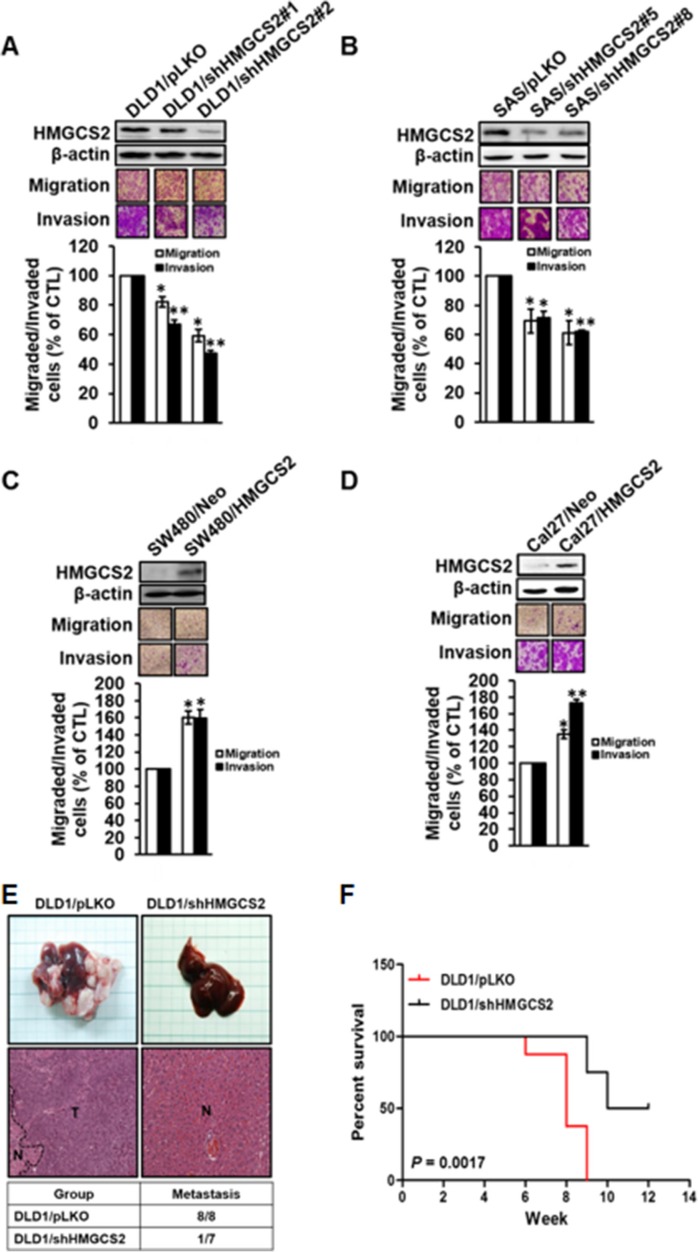
Stable knock-down of HMGCS2 moderates cell migration and invasion ability in CRC and OSCC cells (**A**) Migration and invasion ability of DLD1/pLKO and DLD1/shHMGCS2 was measured with the Boyden chamber assay. Cell migration and invasion toward the lower face of the filter were observed and quantified (lower left panel; *P < 0.05). (**B**) Migration and invasion ability of SAS/pLKO and SAS/shHMGCS2 was measured with the Boyden chamber assay. Cell migration and invasion toward the lower face of the filter were observed and quantified (lower right panel; *P < 0.05). (**C**) Migration and invasion ability of SW480/Neo and SW480/HMGCS2 was measured with the Boyden chamber assay. Cell migration and invasion toward the lower face of the filter were observed and quantified (lower left panel; * *P* < 0.05). (**D**) Migration and invasion ability of Cal27/Neo and Cal27/HMGCS2 was measured with the Boyden chamber assay. Cell migration and invasion toward the lower face of the filter were observed and quantified (lower right panel; *P < 0.05). (**E**) Mice were injected with DLD1/pLKO (*n* = 8) and DLD1/shHMGCS2 (*n* = 7) in the spleen and their livers were excised at the days indicated. (**F**) Survival curves of overall survival in the hepatic animal model.

To examine the direct effects of HMGCS2, we established stable transfectants with HMGCS2 knockdown in CRC cells, including DLD1/shHMGCS2#1 and #2, and OSCC cells, including SAS/shHMGCS2#5 and #8 (Figure 3A, 3B, upper panel). In functional analysis, stable shHMGCS2 transfectants showed significantly reduced migration and invasion ability compared with pLKO control clones (Figure 3A, 3B, lower panel). By contrast, cell motility in SW480 and Cal27 was significantly increased in stable, overexpressed HMGCS2 transfectants (Figure 3C, 3D). Moreover, no difference in proliferation abilities was observed in stable shHMGCS2 or HMGCS2 transfectants (Supplementary Figure 3). From these results, we conclude that HMGCS2 acts as an oncogene, enhancing CRC and OSCC migration and invasion.

To confirm that HMGCS2 is required for *in vivo* metastasis, DLD1/shHMGCS2 transfectants were injected into the spleens of severe combined immunodeficient mice, which were subsequently evaluated for liver metastasis. As demonstrated in Figure 3E (upper panel), less liver metastasis and neoplasms were observed in the DLD1/shHMGCS2 group, compared with the DLD1/pLKO control. As shown in the lower panel, a significant reduction in hepatic metastasis ability was observed in DLD1/shHMGCS2 clones, compared with the pLKO controls (metastatic rate: DLD1/pLKO: DLD1/shHMGCS2 = 100%: 14.28%). Furthermore, knocked-down HMGCS2 expression prolonged survival rates in the hepatic spontaneous metastatic animal model (*P* = 0.0017, Figure 3F). These data suggest that HMGCS2 may be crucial for cell metastasis and that it may reduce mouse survival *in vivo*.

### Ketogenesis activity of HMGCS2 is not involved in OSCC and CRC cell migration and invasion

**Figure 4:**
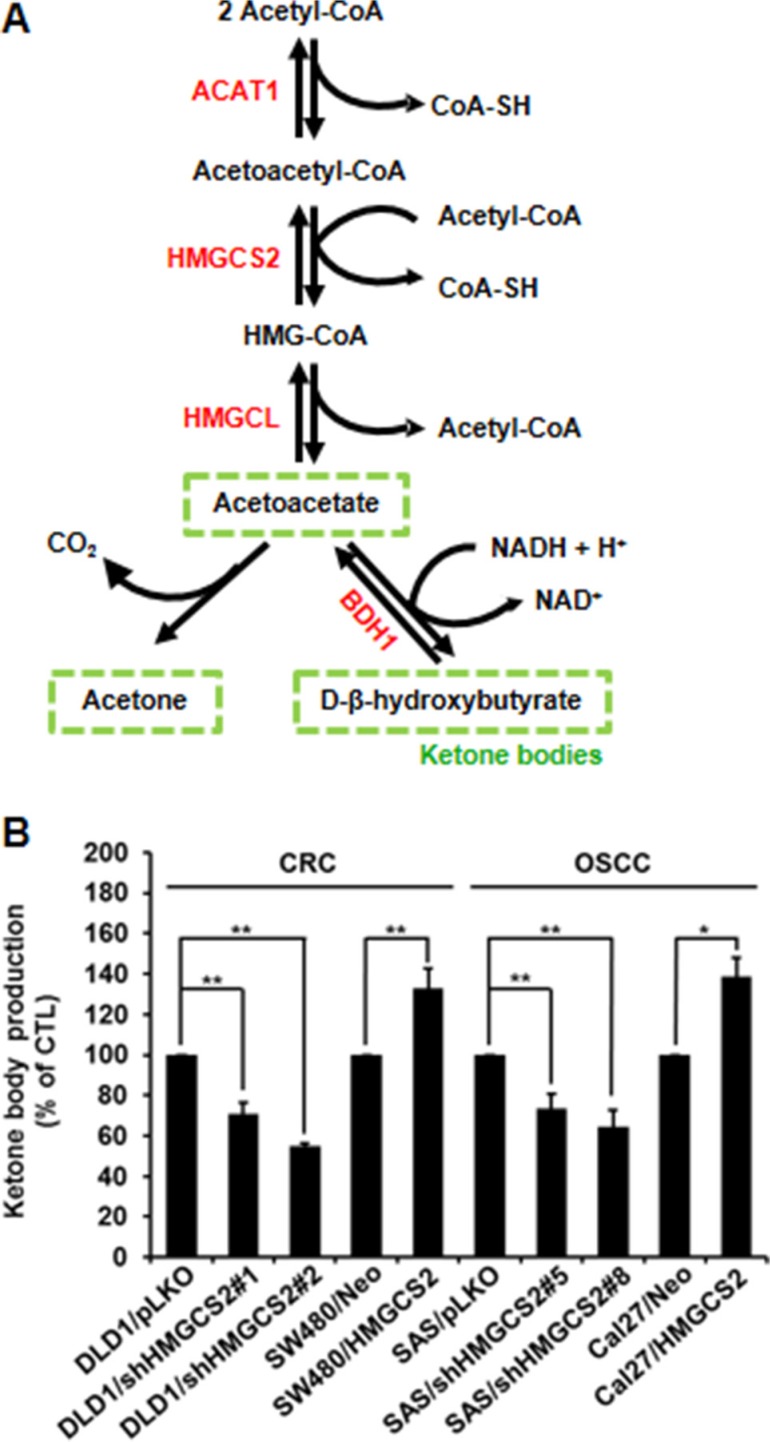

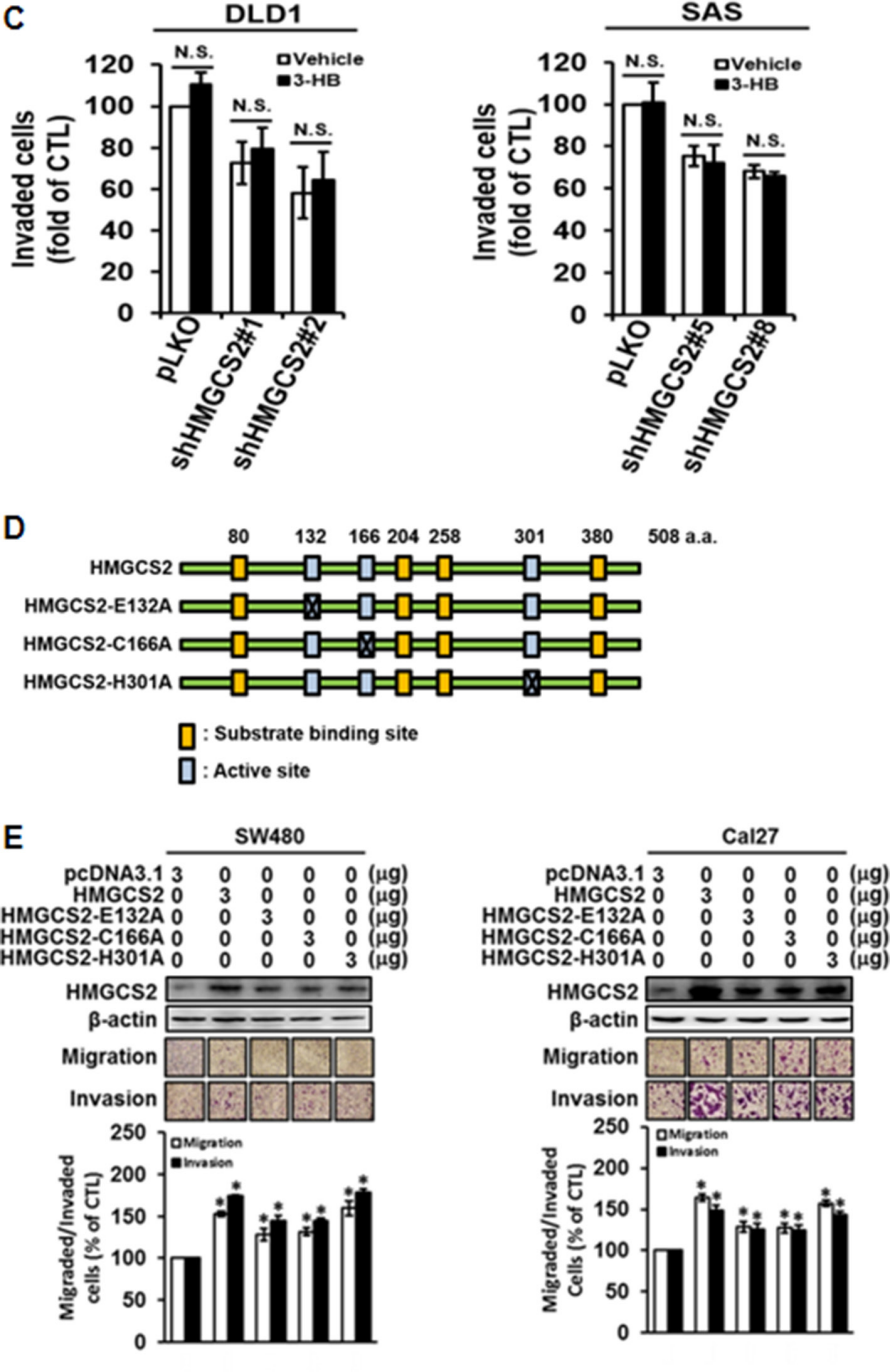
Ketogenesis activity does not affect cancer progression in HMGCS2 transfectants (**A**) Diagram of ketogenesis. (**B**) Ketone body assay was used for the quantitative determination of 3-HB in stable HMGCS2/shHMGCS2 transfectants. (**C**) DLD1/pLKO, DLD1/shHMGCS2, SAS/pLKO, and SAS/shHMGCS2 cells were treated with 3-HB and evaluated for cell invasion by the Boyden chamber assay. The quantification of migratory cell number of DLD1/pLKO and DLD1/shHMGCS2 was conducted in a transwell (lower left panel; *P < 0.05). (**D**) Nucleotide sequence of the HMGCS2 with substrate binding site and active site. (**E**) Cells were transiently transfected with control plasmids or nonenzymatic HMGCS2 expression plasmids. The Boyden chamber assay was used to evaluate the migration and invasion ability and the number of migratory cells in SW480 and Cal27 after transient transfection with nonenzymatic HMGCS2 expression plasmids. The migratory cell number in SW480 and Cal27 cells treated with nonenzymatic HMGCS2 expression plasmids was quantified (*P < 0.05).

Recent reports have indicated that metabolic reprogramming in tumors is a critical factor in cancer progression. HMGCS2 is the rate-limiting enzyme that controls 3-hydroxybutyrate (3-HB) synthesis, the first reaction in ketone body formation (Figure 4A). Therefore, we next investigated whether the ketogenic catalytic activity of HMGCS2 is involved in cell motility. Figure 4B indicates that 3-HB secretion was significantly increased in HMGCS2-stable transfectants and reduced in shHMGCS2 clones. These data indicate that HMGCS2 transfectants may functionally reflect ketogenesis activity. After the addition of the downstream metabolite, 3-HB in shHMGCS2 stable transfectants did not restore invasion (Figure 4C) or proliferation activity (Supplementary Figures 4, 5). To further confirm whether the ketogenic sites of Glu132, Cys166, and His301 in HMGCS2 are crucial in the stimulation of cell motility [22], we established various mutant constructs that lacked enzymatic activity including E132A, C166A, and H301A (Figure 4D). We transfected these plasmids into SW480 and Cal27 cells for 48 h. Figure 4E (left and right panels) shows that overexpression non-enzymatic activity HMGCS2 did not increased cell motility in SW480 and Cal27 cells. Taken together, these results demonstrate that HMGCS2 enhanced cell migration and invasion ability in a ketogenesis-independent manner.

### Identification of Src as the major downstream effector in HMGCS2-enhanced CRC and OSCC progression

**Figure 5:**
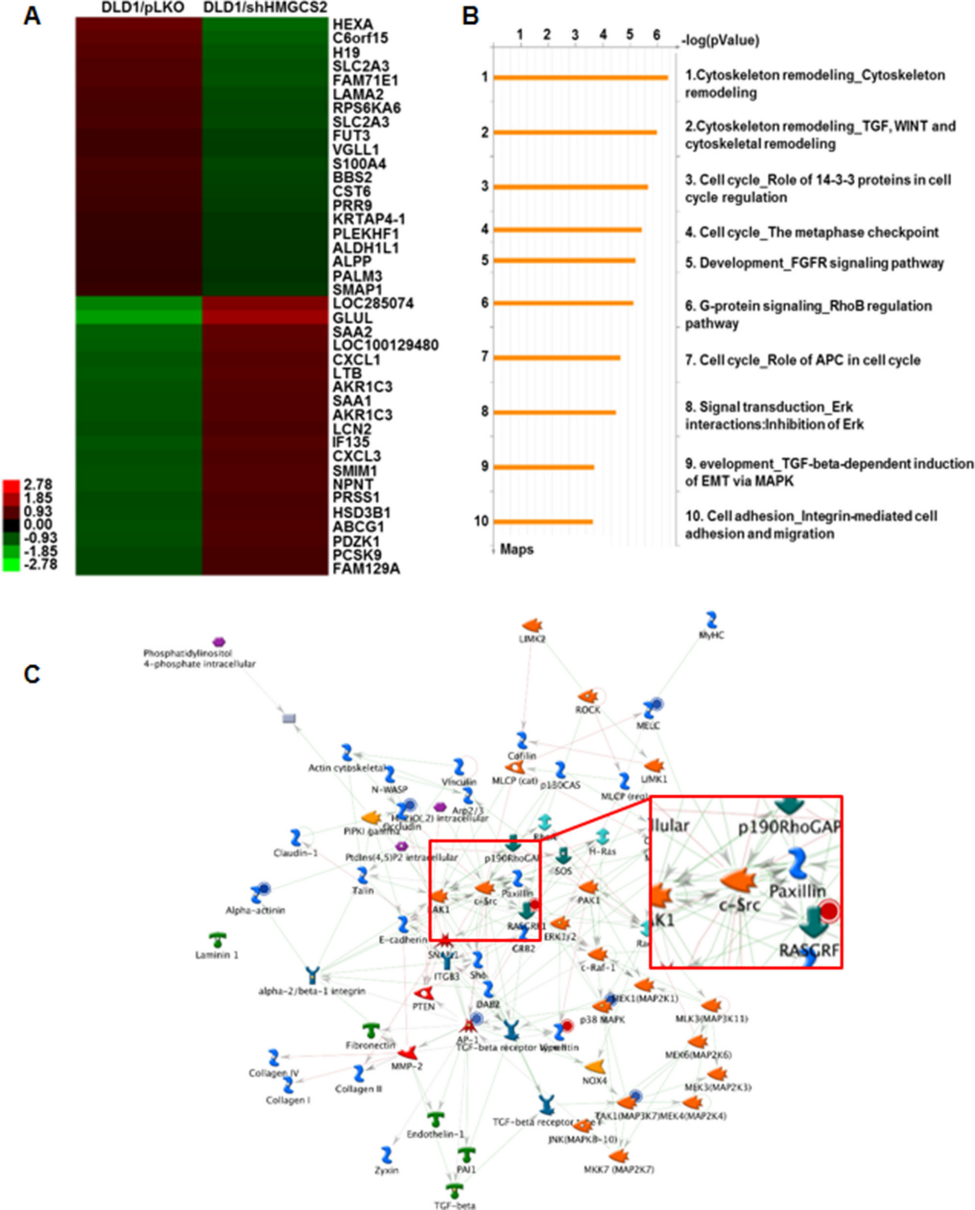

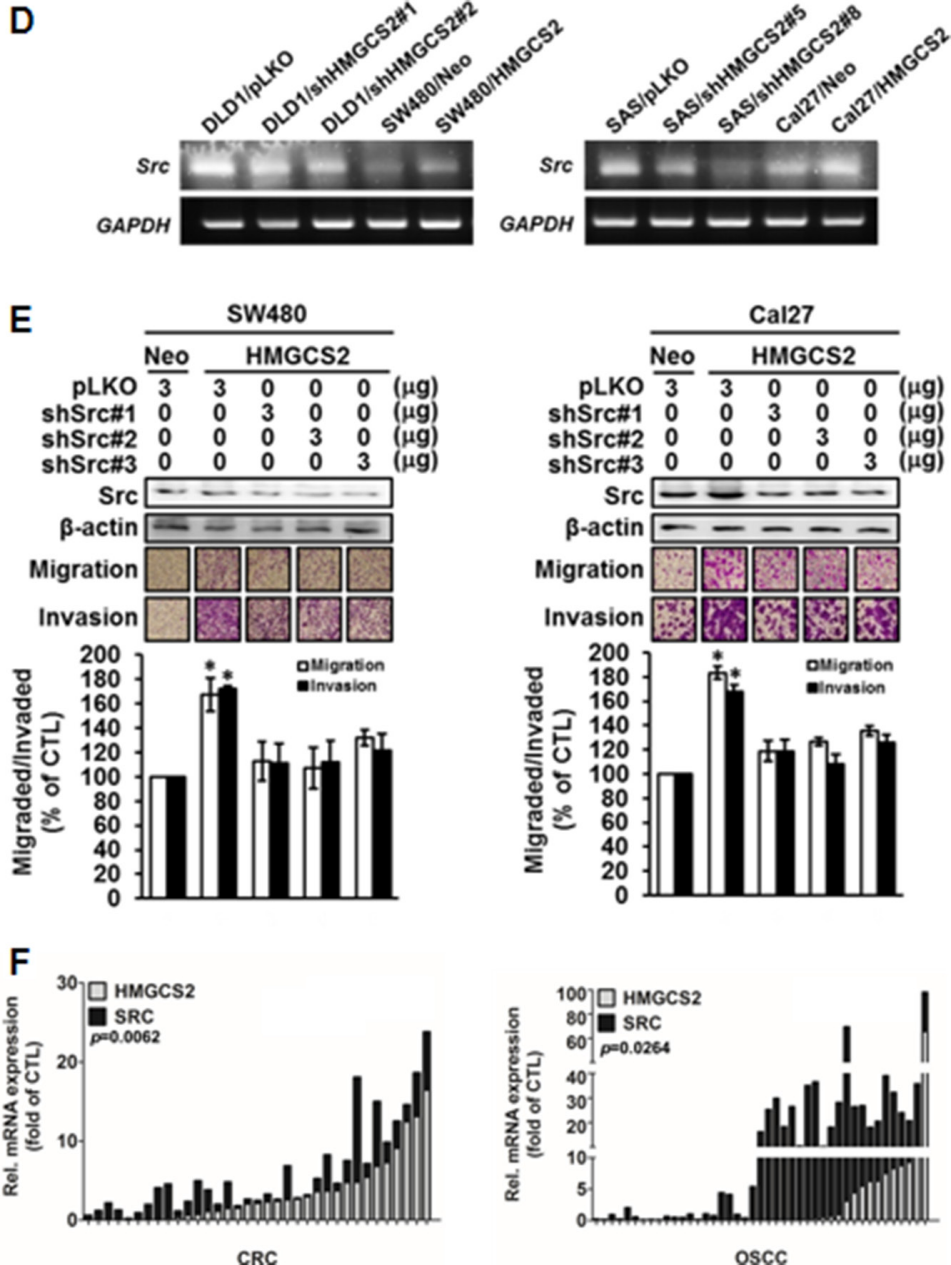
Src plays a role as an essential downstream effector in HMGCS2-induced cancer cell motility (**A**) Heatmap of the mRNA expression profile in DLD1/pLKO and DLD/shHMGCS2 stable clones. (**B**) GeneGo pathway maps. Canonical pathway maps represent a set of approximately 650 signaling and metabolic maps covering human biology (signaling and metabolism) comprehensively. All maps are drawn from scratch by GeneGo annotators and manually curated and edited. The height of the histogram corresponds to the relative expression value for a particular gene/protein. (**C**) Top-scoring network from DLD1/shHMGCS2 versus DLD1/pLKO. Thick cyan lines indicate the fragments of canonical pathways. Upregulated genes are marked with red circles, whereas downregulated genes are marked with blue circles. The “checkerboard” color indicates mixed expression for the gene between files or between multiple tags for the same gene. (**D**) RT-PCR analysis of *Src* expression in stable HMGCS2/shHMGCS2 transfectants. *GAPGH* was used as an internal control for RNA quantity. (**E**) Cells were transiently transfected with control plasmids or various dosages of shSrc expression plasmids (upper panel). The Boyden chamber assay was used to evaluate the migration and invasion ability and number of migratory cells in SW480/HMGCS2 and Cal27/HMGCS2 after transient knockdown of Src. The migratory cell number in SW480/HMGCS2 and Cal27/HMGCS2 cells treated with shSrc expression plasmids was quantified (lower panel; *P < 0.05). (**F**) Quantitative RT-PCR analysis was performed to detect *HMGCS2* and *Src* mRNA expression in CRC and OSCC patients. The data are shown as Log10 of relative quantification, and B2M was used as an endogenous normalization control.

To clarify the downstream effector of HMGCS2 in CRC and OSCC progression, an mRNA microarray study of shMGCS2 transfectants was performed (Figure 5A). GeneGo pathway analysis revealed that cytoskeleton remodeling was at the top scale of the canonical pathway (Figure 5B), further supporting the possibility of HMGCS2 controlling cancer cell motility. Bioinformatic analysis showed that *Src* was the pivotal gene in the shHMGCS2 clone, compared with the pLKO control (Figure 5C). We further found that compared with respective controls, *Src* mRNA expression was significantly reduced in DLD1/shHMGCS2#1, #2 and in SAS/shHMGCS2#5, #8, and that it was increased in SW480/HMGCS2 and Cal27/HMGCS2 clones (Figure 5D). At the functional level, Figure 5E demonstrates that transiently knocked-down Src expression in shSrc plasmids (#1, #2, and #3) in HMGCS2-stable transfectants could reinhibit the rate of migration and invasion in SW480/HMGCS2 (left panel) and Cal27/HMGCS2 (right panel) cells. We then evaluated the clinical association between *HMGCS2* and *Src* mRNA expression in CRC and OSCC patients. Figure 5F shows a positive correlation between *HMGCS2* and *Src* mRNA expression in CRC (left panel, *P* = 0.0062) and OSCC (right panel, *P* = 0.0264) patients. Altogether, these observations further support that the *Src* gene is involved in HMGCS2-enhanced cell motility as a crucial effector *in vitro* and *in vivo*.

### HMGCS2 interacts with PPARα to enhance *Src* expression through a PPARα-binding site on the Src promoter

**Figure 6:**
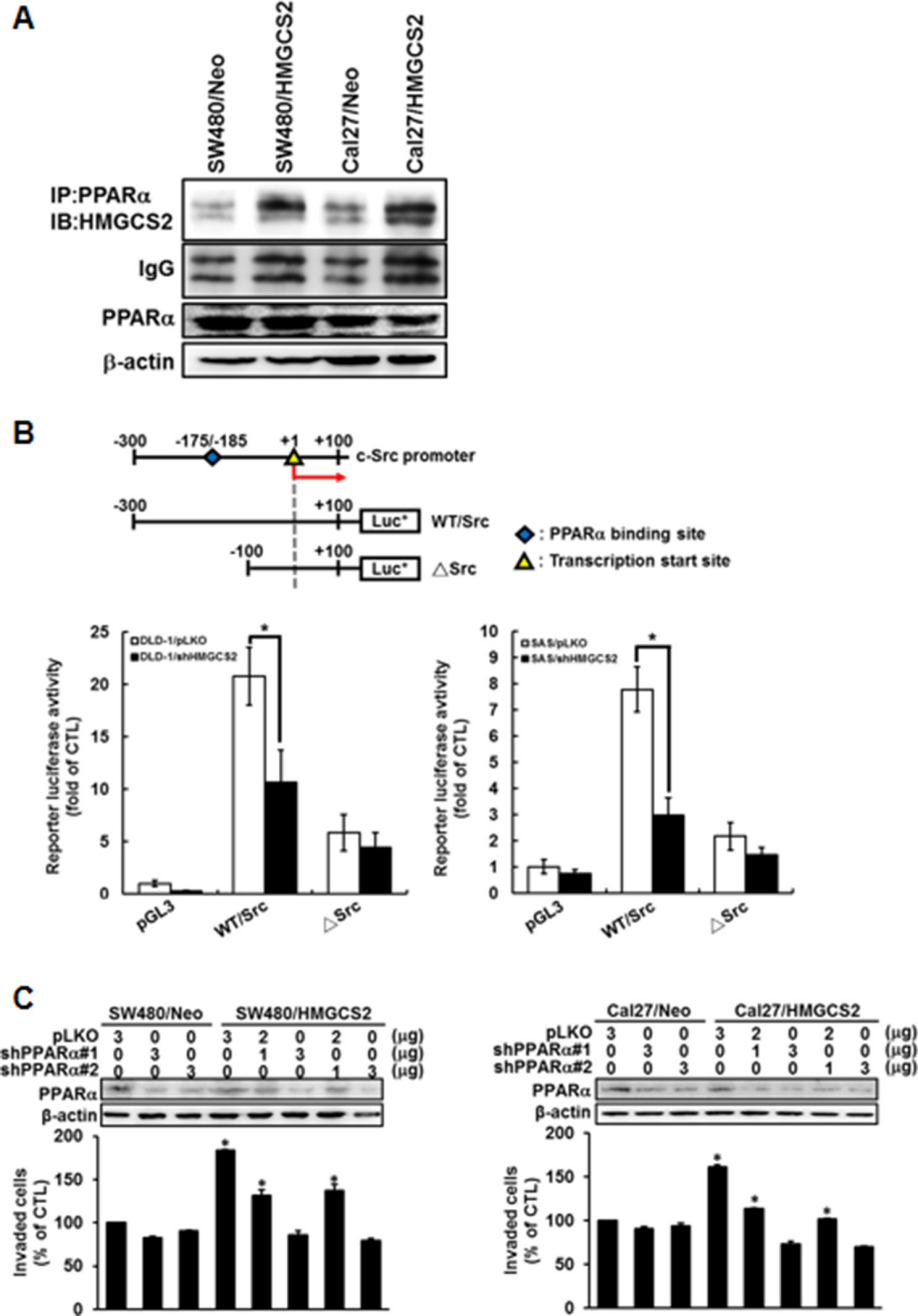

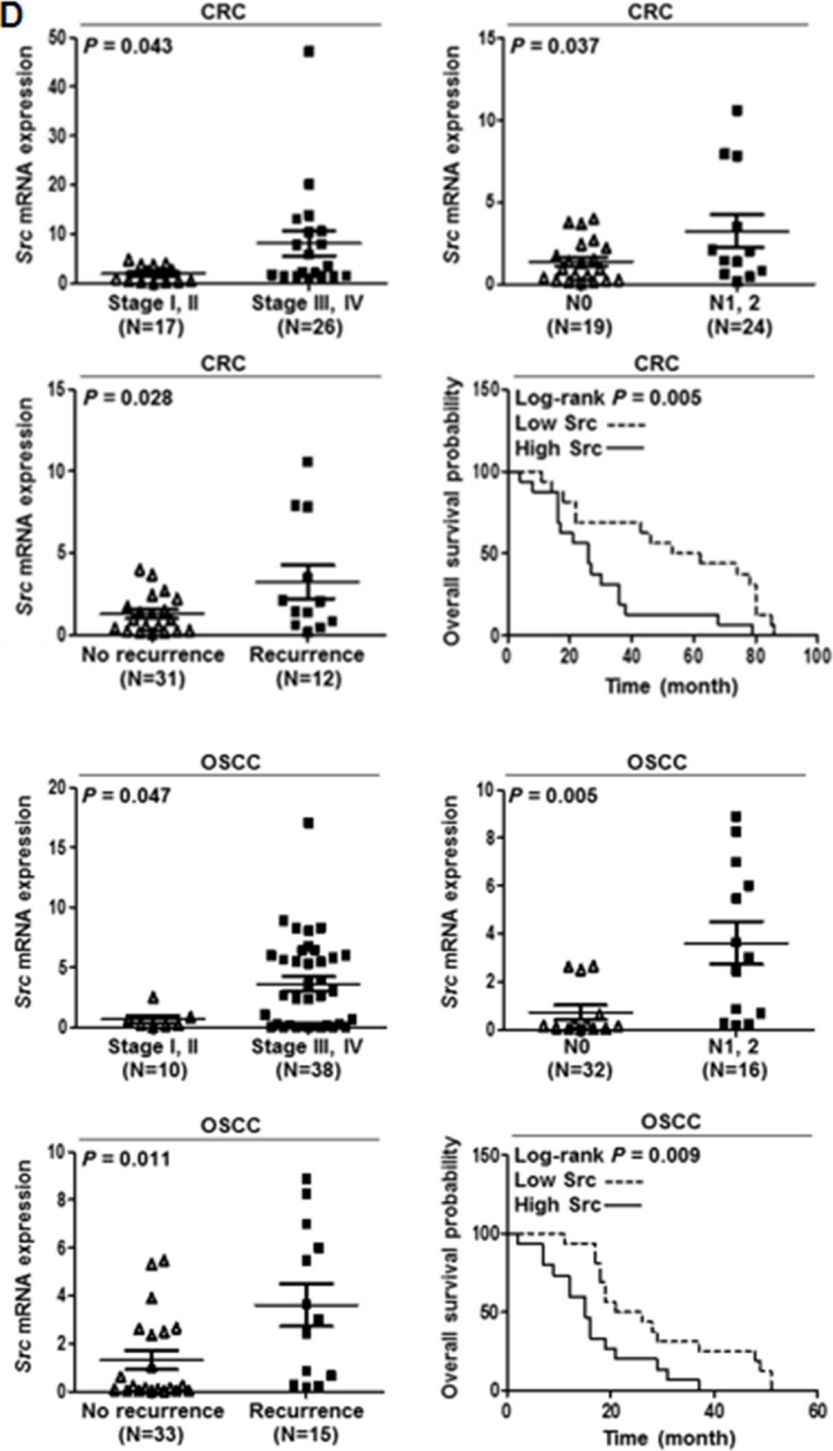
PPARα is a transcriptional co-activator of HMGCS2's up-regulation of Src expression in CRC and OSCC cells (**A**) Whole-cell lysates of SW480/Neo, SW480/HMGCS2, Cal27/Neo, and Cal27/HMGCS2 were prepared for immunoprecipitation with anti-PPARα followed by immunoblotting with anti-HMGCS2. PPARα was used for the internal control. (**B**) Schematic representation of the Src promoter design used in the dual luciferase reporter assay. DLD1/pLKO, DLD1/shHMGCS2, SAS/pLKO, and SAS/shHMGCS2 cells were transiently cotransfected with various promoter constructs for 48 h. Luciferase activity was normalized for transfection efficiency and cell numbers against thymidine kinase (TK) activity from cotransfected TK plasmids. Results are expressed as the mean ± SD, and each experiment was performed three times in duplicate. (**C**) Cells were transiently transfected with control plasmids or various dosages of shPPARα expression plasmids (upper panel). The Boyden chamber assay was used to evaluate the invasion ability and migratory cell number in SW480 and Cal27 after transient transfection with various plasmids. The migratory cell number in SW480 and Cal27 cells treated with HMGCS2 expression plasmids was quantified (lower panel; * *P* < 0.05). (**D**) Real-time quantitative RT-PCR was performed on CRC (*N* = 43) and OSCC (*N* = 48) patients’ tumors. Of the CRC and OSCC patients analyzed, the distributions of demographic, clinical, and pathological features are presented. Patients were divided into high (fold change > cutoff values) or low (fold change ≤ cutoff values) HMGCS2 expression categories. Kaplan–Meier survival curves show that patients with low HMGCS2 expression survived significantly longer than those with high HMGCS2 expression did in both CRC and OSCC patients.

HMGCS2 has been demonstrated to interact with PPARα and to act as a coactivator, upregulating transcription activity from the peroxisome proliferator response element (PPRE) [23]. Moreover, HMGCS2 contains an LXXLL nuclear receptor box and may translocate to a nucleus with PPARα binding, further increasing the transcription of target genes containing PPRE [24–25]. To clarify whether HMGCS2 interacts with PPARα to increase Src transcriptional activity, immunoprecipitation-Western analysis was performed to determine if HMGCS2 could physically interact with PPARα in CRC and OSCC models. Figure 6A shows that the HMGCS2/PPARα complex was more abundant in HMGCS2-stable transfectants than in control clones in both SW480 and Cal27 cell lines. To determine whether HMGCS2 may translocate into nucleus and co-localize with PPARα as well, we performed immunoblots with cytosol and nuclear fraction prepared from SW480/Neo, SW480/HMGCS2, Cal27/Neo, and Cal27/HMGCS2 clones by NE-PER Nuclear and Cytoplasmic Extraction Reagents (Catalog #78833). The equal amounts of cell cytoplasmic (C) and nuclear (N) extracts were blotted for nuclear protein, Lamin B1 (LMNB1) and cytoplasmic probe Glyceraldehyde-3-Phosphate Dehydrogenase (GAPDH). As expected, we demonstrated that HMGCS2 was located both in the cytosol and nucleus, and could physically interact with PPARα as well. Moreover, HMGCS2 transfectants showed more abundant PPARα/HMGCS2 complex compared to Neo control in the nuclear extract of CRC and OSCC cells (Supplementary Figure 6). To be verified the finding endogenously, Supplementary Figure 7 demonstrated that HMGCS2 and PPARα were localize in both cytoplasmic and nucleus. Moreover, HMGCS2/PPARα complex was showed more abundant in the nucleus of DLD1 and SAS cells than SW480 and Cal27 cells (Supplementary Figure 7). Next, we cloned wild-type (WT/Src) and PPARα site deletion (−175/−185 region, Δ*Src*) *Src* promoter constructs (Figure 6B, upper panel), which were then transfected into DLD-1/shHMGCS2 and SAS/shHMGCS2 transfectants. Figure 6B (lower panel) demonstrates that WT/*Src* promoter activity was reduced in the shHMGCS2 clones, compared with the pLKO controls, but no significant difference in the Δ*Src* construct transfection group was observed. These results suggest that HMGCS2 interacted with PPARα to stimulate *Src* promoter activity through the PPARα-binding site in CRC and OSCC cells. We further confirmed whether PPARα is necessary in HMGCS2-induced cell motility. After we transiently transfected silenced-PPARα (shPPARα#1 and #2) expression plasmids into stable overexpressed-HMGCS2 SW480 and Cal27 cells, we determined that the numbers of invaded cells were re-suppressed in a dose-dependent manner in the HMGCS2 transfectants (Figure 6C, left and right panels). To further collect the direct data about the relationship between *Src* mRNA expression and patients’ clinical prognosis, real-time RT-PCR was performed. The result demonstrated that *Src* mRNA expression was positively correlated with CRC and OSCC patients’ TNM stage, lymph node metastases, recurrence probability, and overall survival rate (Figure 6D). Taken together, these results indicate that HMGCS2 physically interacted with PPARα, which was bound to the *Src* promoter, enhancing its transcriptional activity and subsequently increasing OSCC and CRC cell motility and cancer metastasis.

## DISCUSSION

Previous reports have been showed that HMGCS2 may function as an important effector protein in different tumors. However, the clinical relevance and molecular mechanism of HMGCS2 in CRC and OSCC remains unknown. To the best of our knowledge, we firstly identified the *HMGCS2*, a metabolic-related gene, physically interacted with the PPARα protein to regulate Src as a novel mechanism for the promotion of human CRC and OSCC cell migration, invasion and metastasis. First, we provided a strong correlation between the expression of *HMGCS2* and patients’ pathological features in CRC and OSCC. Second, our data demonstrated that HMGCS2 had a critical role in enhanced tumor migration, invasion and metastatic phenotypes through metabolic-independent mechanism *in vitro*. Third, we determined that HMGCS2 interaction with PPARα promoted Src transcriptional activity. These findings suggested that the cooperative relationship between *HMGCS2* and *Src* plays a pivotal role in CRC and OSCC progression and metastasis.

It was well known that cancer cells have unusual metabolic patterns relative to normal cells. Warburg effect is a common example of the intracellular metabolic shift from oxidative phosphorylation to glycolysis. Cumulative evidence suggested that ketone body production and reutilization can drive an increase in tumor growth and metastasis in breast cancer, and several proteins involved in the ketogenesis pathway (HMGCS2, ACAT1, BDH1, HMGCL, and OXCT1) have also been observed to be up-regulated in prostate cancer. In this study, we found that *HMGCS2* was up-regulation in tumor tissue compared with normal tissue, and its expression was strongly correlated with poor clinically outcomes in CRC and OSCC patients. *In vitro* study showed that *HMGCS2* enhanced cancer cell migration and invasion ability in an enzymatic-independent manner. Furthermore, treatment with the downstream metabolic products of HMGCS2 showed no effects in regulating cancer cell motility. Therefore, we provided a novel concept that metabolic role did not appear to be involved in HMGCS2-promoted OSCC and CRC progression.

PPARα was a transcription factor and a major regulator of lipid metabolism in the liver. It was activated under conditions of energy deprivation and necessary for the process of ketogenesis, a key adaptive response to prolonged fasting [28]. Recent work has showed that *PPAR* was a crucial downstream target gene of Ras/Raf/MAPK and extracellular signal-regulated kinase (ERK) pathway in colon cancer [26]. Herein, we identified the subcellular localization of HMGCS2 and PPARα in CRC and OSCC cells. Notably, we found that HMGCS2 could locate both in the cytosol and nucleus, and physically interact with PPARα as well. These data demonstrated that HMGCS2 enhanced cell migration and invasion ability in these two important epithelial type GI track cancers through PPARα signaling.

*Src* was the first and pivotal discovered oncogene, and has essential roles in the regulation of cancer cell invasion, migration, and advanced behavior. Src is the prototypic member of nonreceptor membrane-associated tyrosine kinases, which also includes Fyn, Yes, Blk, Yrk, Fgr, Hck, Lck, and Lyn. When Src is activated, it not only induces cancer cell growth and survival, but also promotes the reorganization of the actin cytoskeleton to invade and reduce cell-cell and cell-matrix adhesion, which ultimately further facilitates motility and invasion. Evidence also reported that Src is an independent indicator of poor clinical prognosis in all stages of human colon carcinoma [27], and could be a biomarker for invasion in OSCC [28]. In our findings, bioinformatic analysis showed that *Src* was the pivotal gene in the shHMGCS2 clone, compared with the pLKO control. Figure 5F showed a positive correlation between *HMGCS2* and *Src* mRNA expression in CRC (left panel, *P* = 0.0062) and OSCC (right panel, *P* = 0.0264) patients. In molecular mechanism, we found that *Src* promoter consists of a nucleotide sequence in the −175/−185 region with potential binding sites for transcription factors including PPARα. Herein, we hypothesize that HMGCS2 interacts with PPARα translocation into nucleus and acts as a coactivator to upregulate the transcriptional activity of Src. As expected, our previous data supports the notion that HMGCS2 physically interacts with the PPARα site on the *Src* promoter, directly activating *Src* promoter activation.

In summary, our study using clinical specimens, cellular experiments, and animal models suggest that HMGCS2 increases cancer cell invasion and metastasis ability via the HMGCS2/PPARα/Src signaling pathway, and plays in a ketogenesis enzymatic-independent manner. Prospectively, *HMGCS2* and its downstream effector(s) could be considered as potential targets for future therapeutic interventions in CRC and OSCC patients.

## MATERIALS AND METHODS

### Study subjects and surgical specimens

Our study included consecutive patients who underwent complete surgical resection for primary CRC and OSCC in National Taiwan University (NTU) Hospital between December 2001 and July 2003. All the surgery was performed by attending surgeons subspecialized to deal with CRC and OSCC, and their clinical and pathologic data were recorded. There was no patient selection bias in this study. Emergency operations for bowel obstruction or perforation and resection for recurrent diseases were excluded in this analysis. Our study was approved by the Institutional Review Board Committee of NTU Hospital.

### Cell lines and cell culture

In this study, seven human colon cancer cell lines (DLD-1, HCT116, LoVo, HT-29, SW620, SW480, and Caco-2) and four human oral cancer cell lines (SAS, CA922, HSC-3, and CAL27) were used. All cells were maintained in Dulbecco's modified Eagle medium (DMEM) (Life Technologies Inc., Carlsbad, CA) with the addition of 4 μM L-glutamine and 10 μM sodium pyruvate (Sigma Chemicals, St. Louis, MO). The cell culture media also contained 10% fetal bovine serum and 1% penicillin (10,000 units/mL) solution. Cells were kept in an incubator with 5% CO_2_ at 37°C. All cells were passaged every 2 to 3 days before confluence.

### Transient transfection and established stable clone cells

The shHMGCS2 plasmids or HMGCS2 construct vectors were transiently transfected into DLD-1, SAS, SW480, and Cal27 cells by using Lipofectamine 2000 transfection reagents (Invitrogen, Carlsbad, California). Briefly, 3 μg of plasmid DNA was mixed with 4.5 μl of transfection reagents. The transfection protocol was performed according to the manufacturer's instructions (Invitrogen, Carlsbad, California) and confirmed by Western blot analysis. After 24 h of transfection, the cells were plated onto fresh media with 10% fetal calf serum and 1000 μg/mL geneticin or 3 μg/mL puromycin. Resistant clones were selected and cultured.

### HMGCS2 overexpression plasmid and point mutation plasmid construction

Total cDNA (complementary DNA) was extracted from the DLD-1 cells, and an HMGCS2 overexpression plasmid was cloned and amplified by PCR with the forward primer 5′-GGTTTCTGCTTG CTCCTCTG-3′ and reverse primer 5′-TATGATTCACG GGG AGAAGC-3′. The resulting fragments were digested with *HindIII* and inserted into a pcDNA3.1 vector. A point mutation plasmid of HMGCS2 was amplified by PCR with primers HMGCS2-E132A (forward: 5′-CTGGAAGTAGG CACTGCGACCATCATTGACAAG-3′; reverse: 5′-CTTG TCAATGATGGTCGCAGTGCCTACTTCCAG-3′), HMGCS2-C166A (forward: 5′-GATACCACCAATGCC GCCTACGGTGGTACTGCC-3′; reverse: 5′-GGCAGTAC CACCGTAGGCGGCATTGGTGGTATC-3′), and HMGC S2-H301A (forward: 5′-GCAAAAGGGTGTAGCAAA GATCATGTACTGTAAATCGTCAAGG-3′; revere: 5′-TG ACGATTTACAGTACATGATCTTTGCTACACCCTTT TGCAAG-3′), with the HMGCS2 expression plasmid serving as a template.

### Src promoter plasmid construction

Total genomic DNA was extracted from the DLD-1 cells, and the *Src* promoter was cloned and amplified by PCR with the forward primer 5′-GGGCATCACCTCATTTCATC-3′ and reverse primer 5′-GTTTGCAAGGCTGGCTTAAA-3′. One deletion promoter of *Src* was generated by deleting the PPARα binding site. A deletion construct was generated with the forward primer 5′-CACTGGGTAAA-3′ in combination with the reverse primer 5′-GTTTGCAAGGCTGGCTTAAA-3′, with the Src promoter serving as a template. The resulting fragments were digested with *HindIII* and *BglII* and inserted into a pGL3 vector.

### Site-directed point mutagenesis protocol

QuikChange II XL Site-Directed Mutagenesis Kit (Catalog #200521) was used to construct point mutation plasmid of HMGCS2. A point mutation plasmid of HMGCS2 was amplified by PCR with primers HMGCS2-E132A (forward: 5′-CTGGAAGTAGGCACTGCGACC ATCATTGACAAG-3′; reverse: 5′-CTTGTCAATGATGG TCGCAGTGCCTACTTCCAG-3′), HMGCS2-C166A (forward: 5′-GATACCACCAATGCCGCCTACGGTGGT ACTGCC-3′; reverse: 5′-GGCAGTACCACCGTAGGC GGCATTGGTGGTATC-3′), and HMGCS2-H301A (forward:5′-GCAAAAGGGTGTAGCAAAGATCATGT ACTGTAAATCGTCAAGG-3′; reverse: 5′-TGACGATT TACAGTACATGATCTTTGCTACACCCTTTTGCAA G-3′), with the HMGCS2 expression plasmid serving as a template. First, add 1 ml of *PfuUltra* HF DNA polymerase (2.5 U/μl) to each control and sample reaction. Add 1 ml of *Dpn* I restriction enzyme (10 U/μl). Gently and thoroughly mix each reaction, spin down in a microcentrifuge for 1 minute, and immediately incubate at 37°C for 1 hour to digest the parental supercoiled dsDNA. Transform 2 μl of the *Dpn* I-treated DNA from each control and sample reaction into separate 45 μl aliquots of XL10-Gold ultra-competent cells.

### RNA isolation

RNA was isolated from colon adenocarcinoma cells by using TRIzol (Invitrogen, Rockville, MD) or paraffin slides through an RNA isolation kit (Qiagen, Duesseldorf, Germany). Reverse transcription was performed using 5 μg of total RNA at a final reaction volume of 20 μL in Moloney murine leukemia virus reverse transcriptase buffer (10 mM dithiothreitol, all 4 deoxynucleoside 5′-triphosphates [dNTPs; at 2.5 mM each], 1 μg of random primer, and 200 U of reverse transcriptase [Promega Corporation]). The reaction mixture was incubated at 65°C for 5 min, and the reaction was terminated by heating at 42°C for 60 min.

### Real-time PCR

Real-time RT-PCR was performed using FastStart Universal Probe master mix (Roche) and amplified using an Eco Sequence detector (Illumina). Commercial primers specific for HMGCS2 (HS00985427-M1) and beta-2-microglobulin (B2M) (HS99999907-M1) were designed by Applied Biosystems (TaqMan^®^ Gene Expression Assays). The results were adjusted using B2M as a control. The experiment was performed three times or more. Relative quality expression was calculated by cancer tissue samples and normal samples, which were chosen as the control group. The differences in values between groups were then measured as fold differences.

### Immunoprecipitation and Western blot analysis

For each immunoprecipitation, cells were lysed in a radioimmunoprecipitation assay (RIPA) buffer (150 mM NaCl, 50 mM Tris base, 5 mM EDTA, 1% NP-40, and 0.25% deoxycholate; pH 7.4). Protein concentrations were determined using the BCA protein assay kit (Pierce, Rockford, IL, USA). Lysates were incubated for 2 h at 41°C with gentle rotation and with rabbit polyclonal antibodies for human PPARα (Aviva, San Diego, USA) immobilized onto protein G magnetic beads (Thermo, Waltham, USA). The beads were washed twice with an RIPA buffer and boiled in a sodium dodecyl sulfate-polyacrylamide gel electrophoresis (SDS-PAGE) sample buffer. Supernatants were immediately subjected to Western blot analysis. For Western blotting, cells were washed twice with ice-cold PBS and collected with a lysis buffer. Proteins (60 μg) were separated by SDS-PAGE on a 4%–15% acrylamide gradient gel (Bio-Rad) and electrophoretically transferred onto polyvinylidene difluoride (PVDF) membranes (Millipore, Bedford, MA). The membranes were blocked in a solution containing 5% nonfat dry milk in PBS-T (0.1% Tween 20, 137 mM NaCl, 10 mM phosphate, 2.7 mM KCl, pH 7.4) and probed with anti-HMGCS2 (Sigma, CA) and anti-β-actin (Sigma, CA) primary antibodies at 4°C overnight. The membranes were then incubated with horseradish peroxidase-conjugated polyclonal secondary antibodies (1:5000) for 1 h. Antibody-protein complexes were detected with enhanced chemiluminescence reagents (Millipore, Bedford, MA), and an image of the blot was documented using the LAS 4000 camera system (Fujifilm, Tokyo, Japan).

### *In vitro* migration and invasion assay

The Boyden chamber is a tool to study cell migration and cell invasion. It consists of a cylindrical cell culture insert nested inside the well of a cell culture plate. The insert contains a polycarbonate membrane at the bottom with a defined pore size. For migration assay, we used permeable Transwells with a pore size of 8 μm and in 24-well dishes (Nucleopore Corp., Pleasanton, CA). Approximately 1 × 10^5^ cells in 100 μL of complete medium were placed in the upper chamber and 1 mL of the same medium was placed in the lower chamber. The cultures were then incubated for 48 h. The cultured cells were subsequently fixed in methanol for 15 min, and the upper side cells on the filters were removed with cotton-tipped swabs. The filters were cleared with PBS, and the remaining cells were stained with 0.05% crystal violet in PBS for 15 min. The underside cells of the filters were viewed and counted using a Leica Microsystems (Type: 090-135.001) microscope. For each experiment, each clone was plated in duplicate and the entire experiment repeated at least three times. For invasion assay, we used the permeable transwell further coated with “matrigel” (40 μg; Millipore) in 24-well dish (Nucleopore Corp., Pleasanton, CA). The matrigel is used to mimic extracellular matrix which is the cellular bio-barrier *in vivo*. When cancer cell invaded, they need to digest the matrigel and invade to the other side of the membrane of Boyden Chamber.

### MTT assay

Cells were prepared in 24-well plates at a density of 5 × 10^4^ cells per well. The cells were then washed twice with PBS and immersed in 0.2 mL MTT reagent (1 mg/mL) per well and incubated for 30 min. The solution was gently removed, and 0.4 mL DMSO, which solubilizes formazan, was added to each well. The cells were then shaken at 30 rpm for 15 min and transferred to 96-well plates. Optical density values were obtained using an ELISA plate reader fitted with a 570 nm filter (Multiskan EX, Thermo).

### 3-HB (ketone body) colorimetric assay

The ketone body assay was based on 3-HB dehydrogenase-catalyzed reactions, in which the change in NADH absorbance, measured at 450 nm, is directly related to the 3-HB concentration of a sample. For the assay, 50 μL of 3-HB standard reagent or sample was loaded onto 96-well plates. Subsequently, 50 μL of development solution was added to each well, and the plates were shaken for 30 min. The optical density value for each well was obtained using an ELISA plate reader fitted with a 450-nm filter (Multiskan EX, Thermo).

### Animal model of hepatic metastasis

Characterization of an animal model of hepatic metastasis was performed as described previously [29]. Colon cancer cell line, DLD1 transfectants (pLKO and shHMGCS2 stable clones) were injected into the spleen of 6-week-old female SCID mice. The mice were sacrificed when they appeared moribund or after 50 % of the group had died. Postmortem examinations included measuring liver metastases and survival rates. These studies were approved by the Institutional Review Board and Institutional Animal Care and Use Committee of National Taiwan University (NTU), and was performed at NTU Hospital.

### Dual luciferase reporter assay

For the dual luciferase reporter assay, 1 × 10^5^ cells were transfected with a reporter vector, renilla luciferase vector, and indicated expression plasmids by using Lipofectamine^™^ 2000 (Invitrogen Corporation, Calsbad, CA). Growth media were removed from cultured cells, which were then rinsed with 1X PBS. The recommended volume of 1X passive lysis buffer was then dispensed into each culture vessel. The culture vessels were then gently shaken for 15 min at room temperature. Each lysate was transferred to a tube or vial, which was then read with a luminometer (Promega Corporation, Madison, WI).

### cDNA microarray

Total RNA was isolated from stable transfectants and their parent clones. Amplification and hybridization were performed according to the manufacturer's protocol (Illumina). Resuspended cRNA samples were dispensed onto BeadChips, and the BeadChips were placed in an Illumina hybridization oven and left to hybridize overnight. The following day, the BeadChips were removed from the oven and their coverseals were removed. The chips were then washed, blocked, stained, and scanned. Sample clustering analysis and raw data filtering (*P* < 0.05) were performed, and quantile normalization was performed on the filtered data, followed by a one-way analysis of variance (ANOVA) to identify significant genes. cDNA microarray chip records have been approved and assigned GEO accession numbers (GSE80641).

### System biology analysis

Lists of significant genes were uploaded from a Microsoft Excel spreadsheet onto DAVID functional annotation tools (http://david.abcc.ncifcrf.gov/tools.jsp) and Metacore 6.13 software (GeneGo pathways analysis) (http://www.genego.com). DAVID functional annotation tool analysis and Metacore 6.0 suite generated maps to describe common pathways or molecular connections between controls and experiments on the lists. Table representations of the molecular relationships between the target proteins were generated using a gene ontology analysis on the basis of processes showing significant (*P* < 0.05) association.

### Statistical analysis

Background data of the low- and high-HMGCS2 expression were compared. Scale variables (expressed as the mean ± standard deviation [SD]) were compared using the Mann–Whitney test and nominal variables were compared using Fisher's exact test. Survival and recurrence data were analyzed using the Kaplan–Meier method. Kaplan–Meier curves were compared by a log-rank test. *P* values were two-sided and the significance level was 0.05. Where appropriate, the data are presented as the mean ± SD. Statistical evaluation of the data was performed with Student's *t* test for simple comparisons between values when appropriate. Variables were retained in the model if the associated two-sided *P* values were 0.10 or lower. All statistical tests were two-sided. *P* values of less than 0.05 were considered statistically significant.

## SUPPLEMENTARY MATERIALS FIGURES


