# Supplementary material for: HMGCS2 enhances invasion and metastasis via direct interaction with PPARα to activate Src signaling in colorectal cancer and oral cancer
Source: Oncotarget. 2016 Nov 1;8(14):22460–76. doi: 10.18632/oncotarget.13006 (PMC5410236; doi:10.18632/oncotarget.13006)
Supplement: Supplementary file 1 [file oncotarget-08-22460-s001.pdf]

HMGCS2 enhances invasion and metastasis via direct interaction with PPARα to activate Src signaling in colorectal cancer and oral cancer

Supplementary Materials

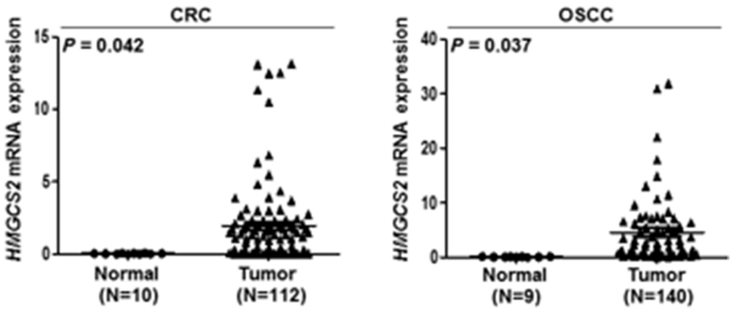

Supplementary Figure S1: *HMGCS2* was highly expression in tumor tissue than normal tissue of CRC and OSCC patients. Real-time quantitative RT-PCR was performed on patients' tumors tissue and adjacent normal samples. *HMGCS2* mRNA level was highly expression in patients' tumor tissue and normal tissue in CRC ($P = 0.042$) and OSCC ($P = 0.037$).

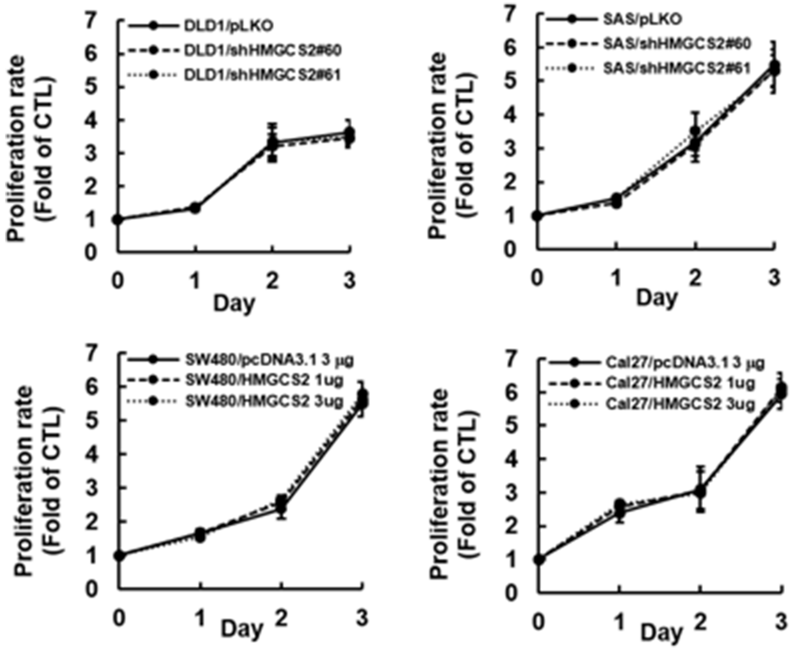

Supplementary Figure S2: HMGCS2 did not affect cell proliferation ability in CRC and OSCC cells. Cells were transiently transfected with control plasmids or various dosages of shHMGCS2/HMGCS2 expression plasmids. The MTT assay was used to evaluate the cell proliferation ability in DLD1, SAS, SW480, and Cal27 after manipulate HMGCS2 expression.

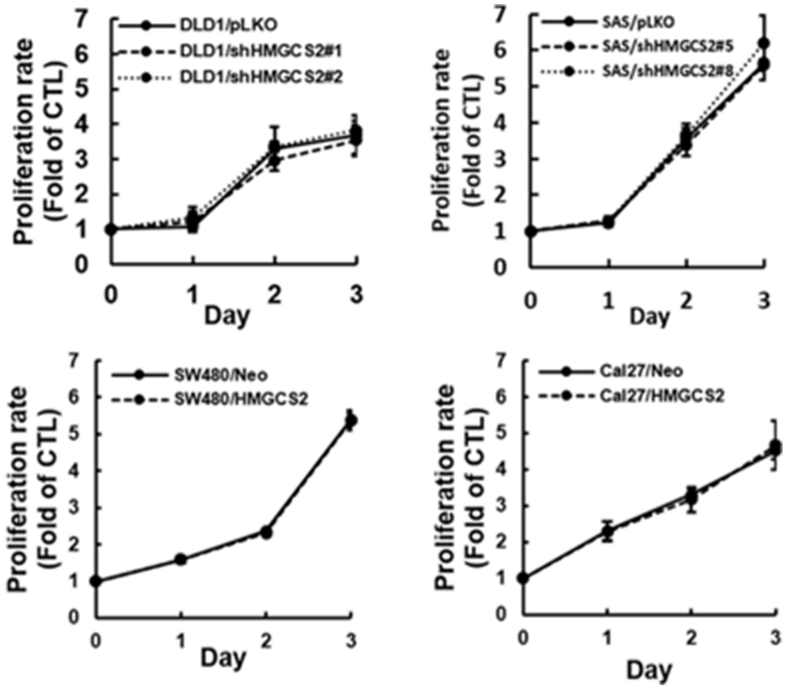

Supplementary Figure S3: HMGCS2 did not affect cell proliferation ability in HMGCS2/shHMGCS2 stable transfectants. Cells were stable transfected with control plasmids or shHMGCS2/HMGCS2 expression plasmids. Resistant clones were selected and cultured. The MTT assay was used to evaluate the cell proliferation ability of shHMGCS2/HMGCS2 stable transfectants.

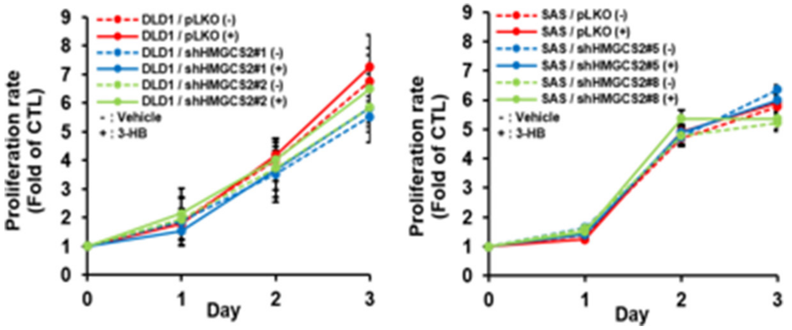

Supplementary Figure S4: 3-HB treatment did not affect cell proliferation ability in HMGCS2 and shHMGCS2 stable transfectants. DLD1/pLKO, DLD1/shHMGCS2, SAS/pLKO, and SAS/shHMGCS2 cells were treated with 3-HB and evaluated for cell proliferation ability by MTT assay.

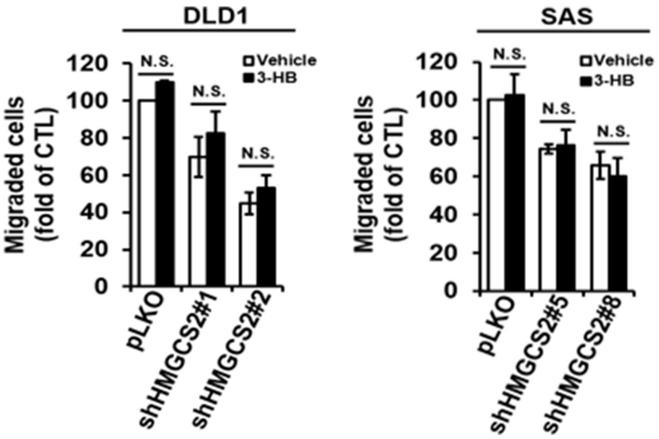

Supplementary Figure S5: 3-HB treatment did not affect cell migration ability in shHMGCS2 transfectants. DLD1/pLKO, DLD1/shHMGCS2, SAS/pLKO, and SAS/shHMGCS2 cells were treated with 3-HB and evaluated for cell migration by the Boyden chamber assay. The quantification of migratory cell number of DLD1/pLKO and DLD1/shHMGCS2 was conducted in a transwell.

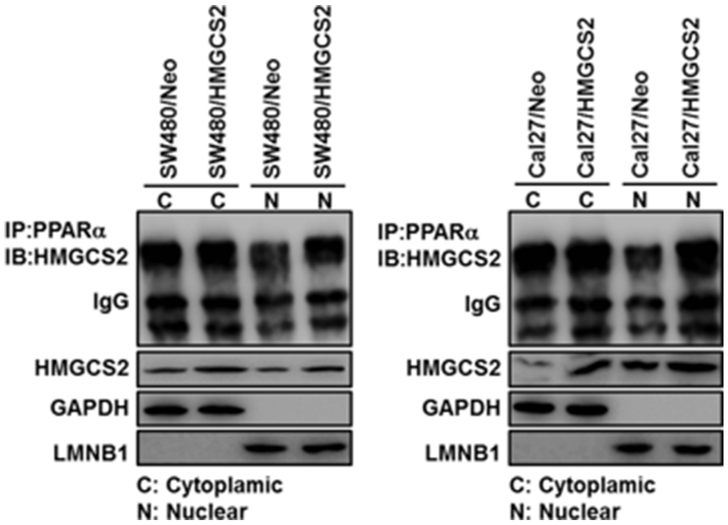

Supplementary Figure S6: HMGCS2 co-localize with PPARα in both cytoplasm and nucleus of HMGCS2 stable transfectants. Cytosol and nuclear fraction prepared from SW480/Neo, SW480/HMGCS2, Cal27/Neo, and Cal27/HMGCS2 clones by NE-PER Nuclear and Cytoplasmic Extraction Reagents. The equal amounts of cell cytoplasmic (C) and nuclear (N) extracts were blotted for nuclear protein, LMNB1 and cytoplasmic probe GAPDH.

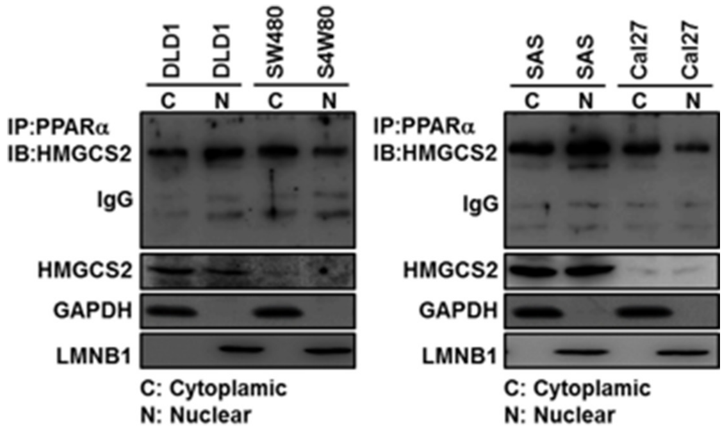

Supplementary Figure S7: HMGCS2 and PPARα were localize in cytoplasm and nucleus of wildtype CRC and OSCC cell lines. Cytosol and nuclear fraction prepared from DLD1, SW480, SAS, and Cal27 cells by NE-PER Nuclear and Cytoplasmic Extraction Reagents. The equal amounts of cell cytoplasmic (C) and nuclear (N) extracts were blotted for nuclear protein, LMNB1 and cytoplasmic probe GAPDH.
